# Preclinical PK investigation of a novel IDO1/TDO dual inhibitor—SHR9146 in mouse plasma and tissues by LC-MS/MS

**DOI:** 10.3389/fonc.2023.1191778

**Published:** 2023-07-26

**Authors:** Mei Xiao, Kan Zhong, Li Guo, Wei Li, Xiaoli Wang, Zhenjun Qiu, Taijun Hang

**Affiliations:** ^1^Department of Pharmaceutical Analysis, China Pharmaceutical University, Nanjing, China; ^2^Research institute, Jiangsu Hengrui Pharmaceuticals Co., Ltd., Lianyungang, China; ^3^Suzhou Haike Medical Technology Co., Ltd., Suzhou, China

**Keywords:** SHR9146, IDO1/TDO inhibitor, pharmacokinetics, tissue distribution, LC-MS/MS

## Abstract

**Purpose:**

The aim of the present study was to establish a liquid chromatography–tandem mass spectrometry (LC-MS/MS) method for the determination of SHR9146, a novel IDO1/TDO dual inhibitor, in mouse plasma and tissues, and to apply it to investigate the preclinical plasma pharmacokinetics and tissue distribution of SHR9146 in mice.

**Methods:**

Samples were spiked with deuterated SHR9146-d_4_ as an internal standard and pretreated by protein-precipitation extraction with methanol. Chromatographic separation was performed on a Venusil ABS C18 column (150 × 4.6 mm, 5 μm) by isocratic elution with 10 mM ammonium acetate buffer containing 0.1% formic acid solution and methanol as mobile phases. MS detection was conducted in positive electrospray ionization with multiple reaction monitoring at *m/z* 444.1/229.4 for SHR9146 and *m/z* 448.4/229.2 for the internal standard.

**Results:**

The method showed good linearity in the calibration range from 0.05 to 50.0 μg/mL. Precisions (intra- and inter-run) were in the range from 0.5% to 5.1%, and accuracies (RE) were between −3.0% and 4.4% for all the concentration levels. SHR9146 was stable in all the tested bio-samples with recoveries >90%. Pharmacokinetic parameters were obtained by non-compartmental analysis. SHR9146 has a half-life of 0.713 h when IV-injected, with CL 12 mL/min/kg and Vd 0.666 L/kg. After oral dosing from 20 to 80 mg/kg, Cmax (range from 8.751 to 12.893 μg/mL) and AUC**_0-t_** (range from 15.606 to 69.971 μg·h/mL) of SHR9146 showed dose proportionality. Other post-oral pharmacokinetic parameters in plasma were as follows (*n*=6): Tmax 0.79 ± 0.36 h, t_1/2_ 1.586 ± 0.853 h, CL 19.8 ± 0.9 mL/min/kg, Vd 3.427± 1.617 L/kg, and absolute bioavailability of 54.2% ± 12.6% (range from 40.2% to 64.7%). In addition, SHR9146 was found to be absorbed rapidly and distributed widely and mainly in the stomach, adrenal gland, liver, and lung.

**Conclusion:**

The method was simple, sensitive, accurate, and specific and was successfully applied for the preclinical pharmacokinetic and tissue distribution study of SHR9146 in mice. The results showed that SHR9146 had dose-independent kinetics in mice via oral administration and was absorbed rapidly and distributed widely. The study provides a good basis for further drug development assessment.

## Introduction

Tryptophan catabolism is closely related to antitumor immune suppression in several types of human cancers (1, 2). Approximately 95% of tryptophan is metabolized through the kynurenine (Kyn) pathway (3). Indoleamine 2,3-dioxygenase (IDO) and tryptophan 2,3-dioxygenase (TDO) are heme-containing enzymes that catalyze the first and rate-limiting step along the Kyn pathway. This process depletes tryptophan and generates tryptophan dioxide, which was converted to *N*-formyl kynurenine and kynurenine derivatives (4).

Tryptophan depletion and kynurenine metabolite accumulation lead to the inhibition of effector T cells and enhancement of regulatory T cells (Tregs) and further induce tumors to resist or escape immune rejection (2, 5–7). IDO and TDO are found to be constitutively overexpressed in a wide variety of tumors (3, 8), and overexpression of IDO1 and TDO is correlated with poor prognosis for survival in cancer patients (8–10). It is also observed that expression of IDO by mice tumor cells prevents their rejection by pre-immunized mice (2). Therefore, IDO1 and TDO, which enable cancer cells to escape from immunologically mediated rejection, become the attractive targets for the development of inhibitors.

At present, development of IDO inhibitors is ongoing in both academic research and pharmaceutical companies, including numerous IDO1 inhibitors and fewer IDO1/TDO dual inhibitors. A number of small-molecule inhibitors are undergoing preclinical study. Several inhibitors, such as epacadostat, BMS-986205, indoximod, navoximod, and PF-0684003, have advanced into clinical trials as single agents or combination with chemotherapeutic agents and immunological checkpoint mediators (11–16). The fast-developed IDO1 inhibitor, epacadostat, could raise response rates and enhance the result of PD-1 inhibitors in phase I/II trials (11, 17). However, in the phase III trial, epacadostat combined with a PD-1 checkpoint inhibitor has no significant improvement in progression-free survival rate compared with that of pembrolizumab alone (18). This result may have an adverse effect on the development of IDO1 inhibitors. However, the potential of IDO inhibitors as anticancer agents to improve cancer immunosuppression is undeniable. The IDO1/TDO dual inhibitors may become a new strategy for tumor immunotherapy.

**Figure 1:**
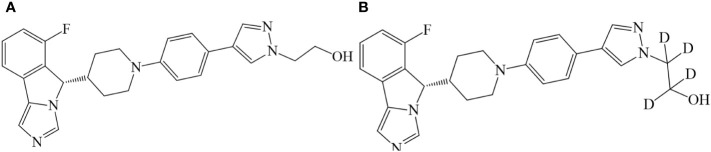
Chemical structures of SHR9146 **(A)** and its d4 substitute (SHR9146-d_4_, **B**).

Based on research of the structure–activity relationship, Jiangsu Hengrui Pharmaceuticals Co., Ltd., designed a series of imidazo isoindole derivatives and reported that the derivatives could be used for the treatment of diseases with a pathological characteristic of the IDO-mediated tryptophan metabolic pathways (19). The compound SHR9146 (shown in **Figure 1**), as one of the above derivatives, combined with the PD-1 antibody, has a significantly better inhibitory effect on colon cancer (MC38) cells than the single PD-1 antibody or SHR9146 and is better than the PD-1 antibody combined with INCB024360 or NLG0919100 (20). It was also reported that SHR9146 plus camrelizumab in combination with/without apatinib demonstrated promising antitumor activity with acceptable safety in patients with advanced solid tumors (21).

To date, very limited information is publicly available on the pharmacokinetics of SHR9146. In this study, a sensitive liquid chromatography–tandem mass spectrometry (LC-MS/MS) method was developed and validated for quantification of SHR9146 in mouse plasma and 18 tissue-related matrices. The pharmacokinetic parameters and tissue distribution results are important for the IND research and development of SHR9146.

## Materials and methods

### Chemicals and reagents

SHR9146 (batch nos. RS011602160514 and RS011602160819, purity 99.2% and 99.4%, water content 4.75% and 2.87%, respectively) and SHR9146-d4 (deuterated SHR9146, shown in **Figure 1**, batch no. SHR161377-001-00, purity 98.8%) were provided by Jiangsu Hengrui Pharmaceuticals Co., Ltd (Lianyungang, China). Methanol and acetonitrile were all of HPLC grade and were purchased from Merck (Darmstadt, Germany). Formic acid (HPLC grade) was provided by Fluka (Seelze, Germany). Ammonium acetate (HPLC grade) was purchased from ROE (Newmark, DE, USA). Ultrapure water was generated by a Millipore Milli-Q Gradient Water Purification System (Molsheim, France). Other reagents were of analytical grade.

### Analytical instruments

The LC-MS system consists of an Agilent 1200 chromatograph (Agilent, USA, equipped with a G1322A degasser, G11312B pumps, G1367C autosampler, and G1316A column oven) and an API 4000 triple quadrupole mass spectrometer (Applied Biosystems, USA, equipped with an atmospheric pressure chemical ionization source). Data analysis was performed on Analyst 1.6.3 software (Applied Biosystems, USA).

### LC-MS/MS conditions

Chromatographic separation was achieved by using a Venusil ABS C18 column (150 × 4.6 mm, 5 μm, Agela Technologies) protected by the C18 pre-column (4.0 × 3.0 mm, Phenomenex, Torrance, USA) at 40°C with an isocratic mobile phase (methanol: 10 mM ammonium acetate solution containing 0.1% formic acid) (85:15, v/v) at a flow rate of 0.8 mL/min.

The SHR9146 and IS were running in the positive ionization mode detected in multiple reaction monitoring (MRM). The optimized parameters were as follows. The MRM m/z transitions were 444.1/229.4 for SHR9146 and 448.4/229.2 for IS. The de-clustering potential (DP) and collision energy (CE) were 100 and 35 V for SHR9146 and 100 and 36 V for IS, respectively. The curtain gas (CUR) pressure was 172 kPa. The collision gas (CAD) pressure was 55 kPa. The temperature (TEM) was 500°C. The ion source gas 1 (N_2_) pressure was 345 kPa. The dwell time was 200 ms.

### Stock and working solutions

SHR9146 around 10 mg was accurately weighed and dissolved in 400 μL of DMSO and then diluted with MeOH to 10.0 mL as stock solution I. Quality control working solutions at 800, 100, 3, and 0.1 μg/mL were prepared by serial dilution of this solution with methanol, respectively. The internal standard (IS) stock and working solutions were prepared in the same way (1 μg/mL for plasma and 0.2 μg/mL for tissue sample). All the solutions were stored at 4°C.

### Study design

ICR mice with body weight 16–26 g were supplied by Shanghai Lingchang Biotechnology Co., Ltd. (animal breeding license number SCXK (Shanghai) 2013-0018) and housed at Shanghai Institute of Materia Medica, Chinese Academy of Sciences [animal use license number SYXK (Shanghai) 2015-0027]. The mice used in this study were fasted overnight for 12 h but had free access to water. Food was supplied 4 h post-dose. All experiments in this study were conducted in accordance with the Guidelines for the Care and Use of Laboratory Animals of Shanghai Institute of Materia Medica, Chinese Academy of Sciences.

A total of 30 mice were randomly assigned into five groups with equal numbers, males and females. One group was intravenously administrated with 5 mg/kg SHR9146 dissolved in 0.5% DMSO plus 95% saline (adjusted pH to 4.0) under sterile conditions. The remaining four groups were administered respectively with a single dose of 20 , 40, or 80 mg/kg or multiple doses of 20 mg/kg twice daily for 7 days, by oral gavage of SHR9146 dissolved in 0.5% CMC-Na. Blood samples were obtained from the fossa orbitalis vein at 15 and 30 min and 1.0, 2.0, 4.0, 6.0, 8.0, 10.0, and 24.0 h after oral gavage administration. An additional 5-min time point was included for iv injection, as well as the pre-drug administration time points on the fifth, sixth, and seventh days in the multiple-dose group. The blood samples were collected in EDTA K2 anticoagulant blood collection tubes and centrifuged at 1,000 rpm for 5 min at 4°C. The supernatant plasma samples were thus produced.

Another set of 30 mice were randomly assigned into five groups with equal numbers, males and females, for tissue distribution study with oral gavage at a single dose of 40 mg/kg at predetermined different time points: pre-dose and post dose at 1, 3, 6, and 24 h, respectively. The whole brain, heart, liver, spleen, lung, kidney, bladder, pancreas, testis, ovary, uterus, stomach, small intestine, adrenal gland, skin, skeletal muscle, thyroid gland, and abdominal fat tissues as well as the whole blood and plasma samples were harvested. Tissue samples were washed with 0.9% sodium chloride solution to remove the residual blood or contents, blotted on a filter paper, and stored at −70°C prior to the analysis.

### Sample preparation

Protein precipitation was selected for the pretreatment of both the plasma and the tissue homogenate prepared after thawing at room temperature, cut into pieces, and homogenized with a quintuple methanol–water mixture (1:1, v/v) for each tissue sample.

Briefly as follows, an aliquot of 25-μL plasma or tissue homogenate was spiked with 100 μL of internal standard solution (1 μg/mL for plasma, 0.2 μg/mL for tissue homogenate) and 300 μL of methanol added and vortex mixed for 60 s. The mixture was then centrifuged at 14,000 rpm at 4°C for 5.0 min, and the supernatant was diluted with methanol–water (4:1, v/v) in a ratio of 1:9 for the plasma or 1:1 for the tissue homogenate, and 5 μL of the resulting solution was injected for the LC-MS/MS analysis.

### Calibration standards and quality control samples

The plasma calibration standards of 50, 30, 10, 3, 1, 0.3, 0.1, and 0.05 μg/mL were prepared by serial diluting of the SHR9146 stock solution I with blank plasma in a ratio of 1:19 (v/v), respectively. Quality control (QC) samples of 40 (HQC), 5 (MQC), 0.15 (LQC), and 0.05 (LLOD) μg/mL were obtained by diluting quality control working solutions in the same way.

The tissue homogenate calibration standards of 50, 30, 10, 3, 1, 0.3, 0.1, and 0.05 μg/g and quality control (QC) samples of 40 (HQC), 5 (MQC), 0.15 (LQC), and 0.05 (LLOD) μg/g were prepared by sequential diluting of the SHR9146 **stock solution I** with blank tissue homogenate, respectively.

All the calibration standards and the QC samples were divided and packed in 1.5-mL centrifuge tubes and stored at −70°C before analysis.

### Bioanalytical method validation

The SHR9146 mouse plasma and tissue homogenate sample determination method was validated according to the relevant guidelines and requirements for bioanalysis and methods reported in the literature (22) with full validation for plasma and partial validation for the liver tissue homogenate.

#### Selectivity

The selectivity was studied for both mouse plasma and liver tissue homogenate. Each was analyzed with a double-blank (no analyte and IS) sample, a single-blank (no analyte) sample, an LLOQ-spiked sample, and an MQC-spiked sample. Plasma samples and liver tissue homogenate after administration of SHR9146 were also compared. The response of interference should not be greater than 20% of the analyte peak at the LLOQ concentration level and should not be greater than 5% of the IS.

#### Calibration curve and lower limit of quantification

Calibration curves for SHR9146 were constructed using weighted quadratic least square regression with 1/x^2^ (x is the concentration of SHR9146, ng/mL for plasma and ng/g for tissue homogenate as the weighting factor), and data were calculated from the peak area of the analyte relative to the IS. The LLOQ (sensitivity) was defined as the lowest concentration on the calibration curve, at which the precision and accuracy should not exceed ±20%.

#### Precision and accuracy

The precision and accuracy were assessed using the QC samples at low, middle, and high concentrations in six replicates, which were prepared and analyzed in three batches. The relative standard deviation (RSD, %) was applied to evaluate the intra- and inter-run precision. An RSD of <15% was considered acceptable. To assess the accuracy, the relative error (RE, %) was calculated. An accuracy of ±15% was considered acceptable.

#### Matrix effect and extraction recovery

The matrix effect was assessed for six individual plasma samples from six individual mice, one hemolyzed plasma sample, and six individual liver tissue homogenates at the low and high concentrations, with three replicates per concentration. Blank mouse plasma and hemolyzed plasma were extracted with methanol and centrifuged. The supernatants were spiked with LQC or HQC solution and internal standard solution to obtain the samples in the matrix. A blank liver tissue homogenate was prepared in the same way as plasma to get the supernatant, which was mixed with the dried QC solution and internal solution to get the samples in the matrix. The matrix factor (MF) of SHR9146 and IS was obtained by calculating the ratio of peak area of the analytes of samples in the matrix to those of neat standard solutions in water for plasma or in methanol–water for the tissue homogenate. Furthermore, the IS-normalized matrix factor was calculated by dividing the matrix factor of SHR9146 by that of the internal standard. It was considered that the matrix effect was significant if the IS-normalized matrix factor was <85% or >115%.

The extraction recovery was investigated at three concentration QC levels (high, middle, low) in plasma. The recoveries of SHR9146 and IS were determined by comparing the peak areas of SHR9146 and IS spiked before pretreatment with those of the spiked after pretreatment.

#### Stability

The stability of SHR9146 was investigated in plasma samples and tissue homogenate samples at the low and high concentration levels of QC samples in triplicate. The plasma samples were examined under different conditions, including at room temperature for 6 h, three freeze–thaw cycles, and at −20°C and  −70°C for 50 days. Post-preparative plasma samples were studied after being placed at room temperature for 24 h. Mouse tissue homogenate samples were assessed at room temperature for 2 h. Samples were considered to be stable if the deviations between measured concentration and the spiked values were within ±15%.

## Results and discussion

### Method development

The reverse-phase HPLC triple quadrupole tandem mass spectrometric method was chosen for the selective, sensitive, and accurate determination of SHR9146 in the plasma samples. SHR161377, the deuterated SHR9146, was selected as the IS based on the analog’s similar chemical, chromatographic, and plasma pretreatment features.

**Figure 2:**
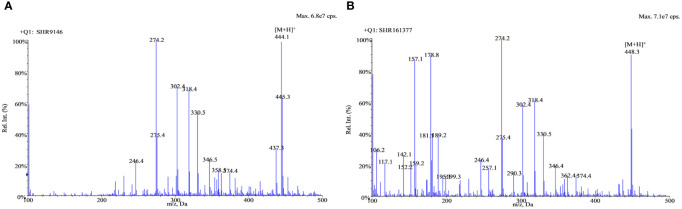
The full-scan spectra of SHR9146 **(A)** and the IS **(B)**.

**Figure 3:**
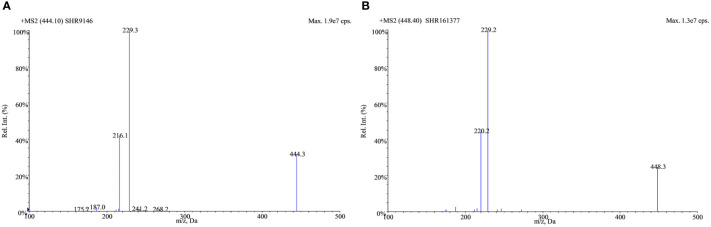
Product mass spectra for the [M + H]^+^ ions of SHR9146 **(A)** and the IS **(B)**.

Positive electrospray ionization was selected for the MS/MS detection since much better sensitivities and stable responses were observed using it than those using negative ionization. The full-scan spectra of SHR9146 and the IS produced predominantly the protonated ions [M + H]^+^ at *m/z* 444.1 and *m/z* 448.4 (**Figure 2**), respectively. The major fragment ions found in their product scan spectra correspondingly were at *m/z* 229.4 and *m*/*z* 229.2 (**Figure 3**). Thus, the MRM acquisitions were made at unit resolution using the ion transitions of *m*/*z* 444.1 → 229.4 for SHR9146 and *m*/*z* 448.4 → 229.2 for the IS.

### Method validation

#### Selectivity

**Figure 4:**
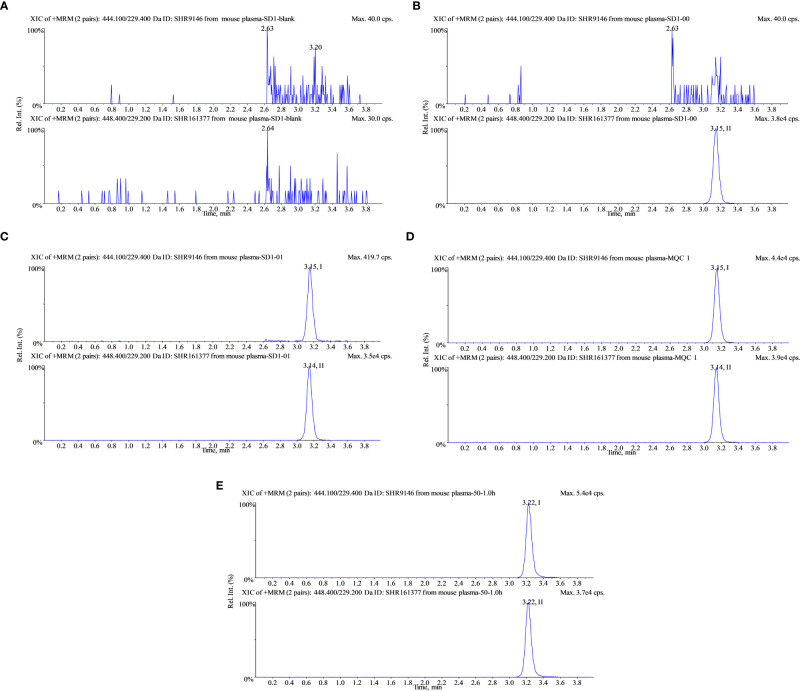
Typical multiple reaction monitoring chromatograms of SHR9146 and the IS in mouse plasma. **(A)** Blank; **(B)** blank spiked with 1 μg/mL IS; **(C)** calibration at 0.05 μg/mL (LLOD) SHR9146 and 1 μg/mL IS; **(D)** calibration at 5 μg/mL (MQC) SHR9146 and 1 μg/mL IS. **(E)** Typical mouse plasma sample after administration of SHR9146. Peak I is SHR9146; peak II is the IS.

The retention time for both SHR9146 and IS was around 3.2 min. Blank responses which were less than 20% of SHR9146 at the LLOQ level and less than 5% of the IS showed that SHR9146 and the IS were not interfered by any endogenous substances in the mouse plasma samples and the liver homogenate (**Figure 4**).

#### Calibration curve

The calibration curve showed a good linear response in concentrations ranging from 0.05 to 50 μg/mL for both the plasma and tissue homogenate. Typical linear correlations between the ratio of peak areas of analyte to IS (y) and analyte concentration (x, μg/mL or μg/g) were y = 0.220 x + 0.0162 (*r* = 0.9997) in plasma (**Figure S1**, **Table S1**) and y = 0.108 x + 0.00102 (*r* = 1.0000) in the liver tissue homogenate (**Figure S2**, **Table S2**).

#### Precision and accuracy

The precision and accuracy of this method were statistically evaluated at three different QC concentration levels and the LLOD level in the plasma and liver tissue homogenate. Assay performance data (*n*=18, 3 runs) of SHR9146 in mouse plasma are shown in **Table 1**. Data (*n*=6) of SHR9146 in the liver homogenate are shown in **Table 2**. The intra-run and inter-run precision and accuracy results at all levels and matrices were within ±15% for high-, medium-, and low-concentration QC samples and within ±20% for LLOQ samples.

**Table 1:** The intra-run and inter-run precision and relative error (*n*=18, 3 runs) and extraction recoveries (*n* =21) of SHR9146 in plasma.

Levels (μg/mL)	SHR9146 (run #1, μg/mL)	SHR9146 (run #2, μg/mL)	SHR9146 (run #3, μg/mL)	Intra-run precision (%)*	mean ± SD (μg/mL)	Inter-run precision (%)	Accuracy (%)	Recovery (%)
40	40.813 ± 0.193	40.829 ± 0.193	40.069 ± 0.331	0.5	40.570 ± 0.433	1.1	1.4	101.0
5	4.917 ± 0.041	5.028 ± 0.057	4.863 ± 0.025	0.8	4.936 ± 0.081	1.6	-1.3	96.8
0.15	0.142 ± 0.003	0.144 ± 0.003	0.157 ± 0.003	1.9	0.147 ± 0.007	4.9	-1.7	94.8
0.05	0.048 ± 0.002	0.049 ± 0.001	0.049 ± 0.001	5.1	0.049 ± 0.002	3.3	-2.8	NA^1^

*Relative standard deviation of SHR9146 in run #1. NA, not applicable.

**Table 2:** The intra-run precision and relative error (*n*=6) in liver tissue homogenate.

Levels (μg/g)	SHR9146 (μg/g)	Intra-run precision (%)	Accuracy (%)
40	38.800 ± 0.190	0.5	−3.0
5	4.945 ± 0.036	0.7	−1.1
0.15	0.1587 ± 0.005	3.0	5.3
0.05	0.052 ± 0.002	4.0	4.4

The extraction recoveries (shown in **Table 1** and **S5**), ranging from 94.8% to 101.0% in mouse plasma at low-, medium-, and high-concentration QC levels, indicated the reliable and efficient sample extraction method established.

#### Matrix effect and extraction recovery

The mean IS-normalized matrix effects at low- and high-concentration QC levels in plasma (**Table S3**) were 99.2 ± 3.6%, 99.4 ± 1.2% and 99.7 ± 1.9%, 99.2 ± 0.6% in the liver tissue homogenate (**Table S4**), respectively, with RSD not more than 2.0%, which indicated that no significant matrix effects were observed.

#### Stability

The results are shown in **Table 3**. It was demonstrated that SHR9146 was stable under various storage conditions. The plasma samples were stable at room temperature for 6 h with the RE being −11.1% and stable after three freeze–thaw cycles with the RE between 0.6% and 1.0%. They were also stable at both −20°C for 50 days and −70°C for 50 days with the RE being 1.2% to −1.6% and −1.2% to 0.5%, respectively. The post-preparative samples were stable at room temperature for 24 h with the RE being 2.8%. The mouse whole blood samples were stable at room temperature for 2 h with the RE being 2.8%.

**Table 3:** Stability of SHR9146 in mouse plasma samples (*n*=3, mean ± SD).

Condition	QC spiked (μg/mL)	Measured value (μg/mL)	RSD (%)	RE (%)
At room temperature for 6 h	0.15	0.133 ± 0.002	1.3	−11.1
	40	35.6 ± 0.17	0.5	−11.1
After three freeze–thaw cycles	0.15	0.151 ± 0.002	1.2	1.0
	40	40.2 ± 0.33	0.8	0.6
At −20 °C for 50 days	0.15	0.148 ± 0.004	2.7	−1.6
	40	39.5 ± 0.11	0.3	−1.2
at -70 °C for 50 days	0.15	0.151 ± 0.004	2.6	0.5
	40	39.5 ± 0.26	0.7	−1.2
Post-preparation at room temperature for 24 h	0.15	0.151 ± 0.003	1.7	0.8
	40	40.3 ± 0.09	0.2	0.8
Whole blood samples at room temperature for 2 h	0.15	0.145 ± 0.002	1.5	−0.5
	40	39.4 ± 0.22	0.5	−0.1

### Plasma pharmacokinetics

The pharmacokinetic parameters of SHR9146 in mouse plasma were estimated by Phoenix 1.4 software using a non-compartmental model.

**Figure 5:**
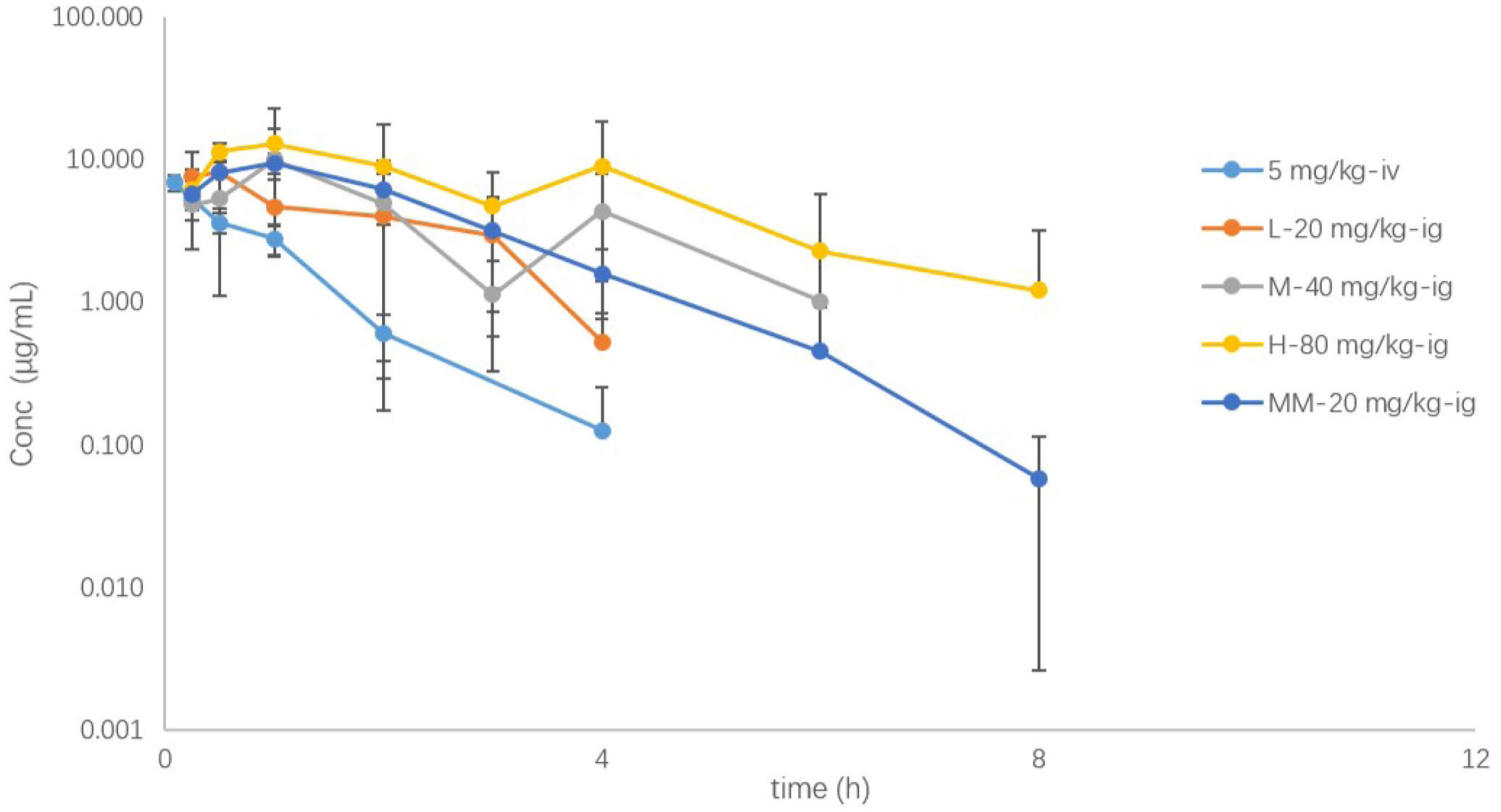
The plasma drug concentration (μg/mL)–time (h) curves of SHR9146 in mice following different doses or routes of administration (*n*=6).

The mean concentration–time curves of SHR9146 in mouse plasma after a single oral gavage dose of 20, 40, or 80 mg/kg, or a single intravenous dose of 5 mg/kg, or the 7th-day steady state after twice-daily multiple oral doses of 20 mg/kg are shown in **Figure 5** (**Table S6**). The main PK parameters are summarized in **Table 4**.

**Table 4:** The plasma pharmacokinetic parameters of SHR9146 in mice (three males and three females).

Dosage (mg/kg)	Gender	*T*_max_ (h)	*C*_max_ (μg/mL)	AUC_0-t_ (μg·h/mL)*	AUC_0-∞_ (μg·h/mL) *	MRT_0-∞_ (h) *	*t*_1/2_ (h) *	CL (mL/min/kg) *	Vd (L/kg) *	F (%) *
20 (i.g.)	Male	0.417 ± 0.144	10.047 ± 2.368	14.4	14.6	1.60	0.531	22.8	2.189	57.7
	Female	0.500 ± 0.433	9.166 ± 4.242	16.8	17.9	1.68	0.866	18.6	1.88	
	Totol	0.458 ± 0.292	9.607 ± 3.111	15.6	16.3	1.64	0.699	20.5	2.02	
40 (i.g.)	Male	1.333 ± 0.577	15.004 ± 3.241	31.0	33.4	2.56	1.66	20.0	3.07	40.2
	Female	1.833 ± 1.893	9.876 ± 3.491	12.4	/	/	/	/	/	
	Totol	1.583 ± 1.281	12.440 ± 4.119	21.7	/	/	/	/	/	
80 (i.g.)	Male	1.833 ± 1.893	15.490 ± 6.147	75.3	75.9	5.90	3.30	17.6	6.22	64.7
	Female	1.167 ± 0.764	18.735 ± 7.301	64.7	65.9	3.29	1.49	20.2	4.00	
	Totol	1.500 ± 1.342	17.112 ± 6.293	70.0	70.9	4.60	2.40	18.8	5.19	
20 (i.g. bid, 7 days)	Male	0.833 ± 0.289	10.486 ± 0.993	24.3	24.4	/	1.06	13.6	/	/
	Female	0.833 ± 0.289	10.879 ± 0.737	24.2	24.3	/	0.951	13.7	/	
	Totol	0.833 ± 0.258	10.683 ± 0.811	24.2	24.4	/	1.01	13.7	/	
5 (i.v.)	Male	/	/	6.73	6.95	0.965	0.761	12.0	0.694	/
	Female	/	/	6.80	6.91	0.882	0.665	12.1	0.638	
	Totol	/	/	6.76	6.93	0.923	0.713	12.0	0.666	

T_max_, time to reach; C_max_, C_max_ peak plasma concentration; AUC, area under curve; MRT, mean residence time; t_1/2_, elimination half-life; CL, clearance; Vd, volume of distribution; F, bioavailability. *Obtained from the average drug concentration–time data for each dose.

SHR9146 reached the maximum concentration in plasma rapidly after oral gavage, with the mean Tmax ranging from 0.458 to 1.583 h. The AUC and Cmax appeared to increase proportionally along with the oral gavage doses ranging from 20 to 80 mg/kg.

After multiple oral doses of 20 mg/kg twice daily for 7 consecutive days, the steady-state Cmax and AUC values were 1.08- and 1.55-fold of those after a single oral dose, respectively. The results indicated that there was no significant accumulation of SHR9146 after multiple-oral-dose administration.

The absolute bioavailability was estimated to be 57.7%, 40.2%, and 64.7% respectively for a single oral dose of 20, 40, or 80 mg/kg, demonstrated with a fair steady absorption through oral administration.

The mean plasma clearance (CL) value was 12.0 mL/min/kg after the intravenous administration, which was equivalent to 13.3% of the blood flow of mouse liver (around 90 mL/min/kg (23)), and the volume of distribution (Vd) was 0.666 L/kg, which was 91.9% of the total body fluid of mice (around 0.725 L/kg (23)). Similar values were observed after the oral gavage doses. The Cl and Vd values were 19.769 ± 0.866 mL/min/kg and 3.427 ± 1.617 μg/mL, respectively, after single dosing ranging from 20 to 80 mg/kg (*n*=6). The mean terminal half-life in plasma exhibited with a low fluctuation range from 0.699 to 1.66 h, except for the high oral dose of 2.40 h.

Therefore, SHR9146 demonstrated linear pharmacokinetics characteristics in mice with oral administration.

### Tissue distribution

After oral administration of 40 mg/kg SHR9146, samples in different tissues at predetermined time points (pre-drug administration, post-drug administration at 1, 3, 6, and 24 h) were collected and prepared for the analysis. The drug concentrations of SHR9146 (μg/g or μg/mL) were determined for each mouse, and the mean and standard deviation were further obtained (**Table S7**).

**Figure 6:**
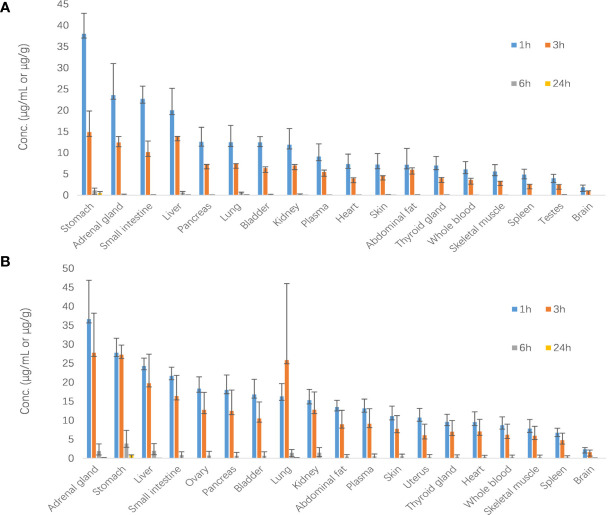
SHR9146 concentrations (μg/g)–time (h) data in different mouse tissues (**A** male *n*=3; **B** female *n*=3).

Results are shown in **Figure 6**. After oral administration of 40 mg/kg, SHR9146 was widely distributed in various tissues and mainly distributed in the stomach, adrenal gland, liver, and lung. The content of SHR9146 in the mouse brain was the lowest. The highest concentrations were found at the 1-h time point for most of the tissues, whereas the lowest concentration at the last time point at 24 h were only around 3.1% of the highest concentration.

## Conclusions

In this study, a sensitive liquid chromatography–tandem mass spectrometry (LC-MS/MS) method was developed and validated for quantification of SHR9146 in mouse plasma and 18 tissue-related matrices. Moreover, it was successfully applied for the plasma pharmacokinetics and tissue distribution study of SHR9146 in mice.

The pharmacokinetics of SHR9146 in mice were determined at an intravenous dose of 5 mg/kg, single oral gavage doses of 20, 40, or 80 mg/kg, and twice-daily multiple oral doses of 20 mg/kg. Pharmacokinetic parameters were estimated by using non-compartmental models. Results showed that both AUC and Cmax were positively correlated with the oral single doses. There was no significant accumulation of SHR9146 in plasma on multiple dosing and no significant difference by route or dose for the pharmacokinetic parameters. The mean terminal half-life in plasma showed a low fluctuation range from 0.699 to 1.66 h, except for the high oral dose of 2.4 h. Oral bioavailability was 54.2% ± 12.6% (range from 40.2% to 64.7%) for single doses. Therefore, SHR9146 has dose-independent kinetics in mice via oral administration based on the above results.

The tissue distribution of SHR9146 in mice were estimated after oral administration of 40 mg/kg. Results showed that SHR9146 was mainly distributed in the stomach, adrenal gland, liver, and lung, whereas it was rarely distributed in the brain owing to the blood–brain barrier of mice. The highest SHR9146 Cmax in plasma and tissue was found within 1 h, indicating that SHR9146 was rapidly absorbed and sampling time points within 1 h should be concerned in preclinical and clinical studies in the future. The Cmax ratio between whole blood and plasma was in the range of 0.6~0.9, indicating that SHR9146 rarely entered red blood cells.

To the best of our knowledge, this is the first reported and successfully validated bioanalytical assay method developed for SHR9146, a novel IDO1/TDO dual inhibitor. Research on pharmacokinetics and tissue distribution also provided further basis for drug–drug interaction studies, dosing regimen, physiologically based PK modelling, and prediction in clinical trials in the future.

## Data availability statement

The datasets presented in this study can be found in online repositories. The names of the repository/repositories and accession number(s) can be found in the article/supplementary material.

## Ethics statement

The animal study was reviewed and approved by Institutional Animal Care and Use Committee of Shanghai Institute of Materia Medica, Chinese Academy of Sciences.

## Author contributions

TH, MX, and KZ conceived and designed the experiments. MX, LG, and WL performed the experiments. MX analyzed the data. MX wrote the original draft. TH, XW, and ZQ supervised the investigation. TH reviewed and edited the manuscript. All authors contributed to the article and approved the submitted version.
