# Supplementary material for: Preclinical PK investigation of a novel IDO1/TDO dual inhibitor—SHR9146 in mouse plasma and tissues by LC-MS/MS
Source: Front Oncol. 2023 Jul 26;13:1191778. doi: 10.3389/fonc.2023.1191778 (PMC10410440; doi:10.3389/fonc.2023.1191778)
Supplement: Supplementary file 1 [file DataSheet_1.docx]

Supplementary Material

Preclinical PK investigation of a novel IDO1/TDO dual inhibitor - SHR9146 in mouse plasma and tissues by LC-MS/MS

Mei Xiao, Kan Zhong, Li Guo, Wei Li, Xiaoli Wang, Zhenjun Qiu and Taijun Hang*

* Correspondence: Prof. Dr. Taijun Hang: hangtj@cpu.edu.cn





Figure S1. Linearity of SHR9146 in mouse plasma samples.

**

**

Figure S2. Linearity of SHR9146 in mouse tissue homogenates.

Table S1. Calibration standards of SHR914 in the mouse plasma

| Concentration (μg/mL) | SHR9146 Area | IS Area | Area ratio |
| --- | --- | --- | --- |
| 0.05 | 2066 | 166884 | 0.0124 |
| 0.1 | 3952 | 169869 | 0.0233 |
| 0.3 | 12461 | 181724 | 0.0686 |
| 1 | 34663 | 159915 | 0.2168 |
| 3 | 119196 | 180228 | 0.6614 |
| 10 | 427376 | 194193 | 2.2008 |
| 30 | 1082212 | 158602 | 6.8234 |
| 50 | 1867913 | 171683 | 10.8800 |

Table S2. Calibration standards of SHR914 in the mouse tissue homogenates

| Concentration (μg/g) | SHR9146 Area | IS Area | Area ratio |
| --- | --- | --- | --- |
| 0.05 | 1392 | 226326 | 0.00615 |
| 0.1 | 2848 | 228312 | 0.01247 |
| 0.3 | 7587 | 224725 | 0.03376 |
| 1 | 23851 | 225779 | 0.10564 |
| 3 | 78303 | 226979 | 0.34498 |
| 10 | 232348 | 221806 | 1.04753 |
| 30 | 730903 | 223550 | 3.26953 |
| 50 | 1193371 | 221334 | 5.39172 |

Table S3. Matrix factors of SHR9146 for mouse plasma at the low(L) and high(H) concentrations (*n*=21) *

| No. | SHR9146 | IS | IS-normalized | No. | SHR9146 | IS | IS-normalized |
| --- | --- | --- | --- | --- | --- | --- | --- |
| 1-L-1 | 96.4 | 103.1 | 93.6 | 1-H-1 | 100.5 | 99.7 | 100.9 |
| 1-L-2 | 94.5 | 106.9 | 88.4 | 1-H-2 | 96.2 | 98.6 | 97.5 |
| 1-L-3 | 103.5 | 100.0 | 103.5 | 1-H-3 | 102.9 | 102.4 | 100.5 |
| 2-L-1 | 100.8 | 102.4 | 98.4 | 2-H-1 | 101.0 | 101.9 | 99.1 |
| 2-L-2 | 103.1 | 100.7 | 102.3 | 2-H-2 | 103.4 | 101.3 | 102.1 |
| 2-L-3 | 101.5 | 98.4 | 103.1 | 2-H-3 | 97.2 | 100.2 | 97.0 |
| 3-L-1 | 102.7 | 101.8 | 100.9 | 3-H-1 | 100.9 | 101.3 | 99.6 |
| 3-L-2 | 100.5 | 100.7 | 99.8 | 3-H-2 | 97.5 | 97.5 | 100.0 |
| 3-L-3 | 98.3 | 99.7 | 98.6 | 3-H-3 | 101.2 | 100.9 | 100.3 |
| 4-L-1 | 103.8 | 101.3 | 102.5 | 4-H-1 | 103.4 | 104.4 | 99.0 |
| 4-L-2 | 102.5 | 100.2 | 102.3 | 4-H-2 | 101.1 | 101.9 | 99.2 |
| 4-L-3 | 98.0 | 102.2 | 95.8 | 4-H-3 | 99.1 | 101.2 | 97.9 |
| 5-L-1 | 106.2 | 105.9 | 100.2 | 5-H-1 | 97.1 | 97.7 | 99.3 |
| 5-L-2 | 97.0 | 96.9 | 100.1 | 5-H-2 | 101.1 | 101.7 | 99.5 |
| 5-L-3 | 96.7 | 101.1 | 95.7 | 5-H-3 | 99.7 | 99.6 | 100.1 |
| 6-L-1 | 102.2 | 104.8 | 97.6 | 6-H-1 | 101.7 | 102.0 | 99.7 |
| 6-L-2 | 100.0 | 100.3 | 99.7 | 6-H-2 | 94.5 | 94.5 | 99.9 |
| 6-L-3 | 102.1 | 102.4 | 99.7 | 6-H-3 | 97.5 | 98.4 | 99.1 |
| G-L-1 | 101.6 | 102.3 | 99.3 | G-H-1 | 103.2 | 105.0 | 98.3 |
| G-L-2 | 100.5 | 102.4 | 98.1 | G-H-2 | 100.6 | 102.0 | 98.7 |
| G-L-3 | 111.4 | 108.3 | 102.9 | G-H-3 | 100.3 | 100.9 | 99.5 |
| mean | 101.1 | 102.0 | 99.2 | mean | 100.0 | 100.6 | 99.4 |
| SD | 3.7 | 2.7 | 3.6 | SD | 2.5 | 2.4 | 1.2 |
| RSD | 3.7 | 2.7 | 3.6 | RSD | 2.5 | 2.4 | 1.2 |

^*^: Low concentration was 0.15 μg/mL; high concentration was 40 μg/mL.

Table S4. Matrix factors of SHR9146 for mouse liver tissue homogenates at the low(L) and high(H) concentrations (*n*=18) ^*^

| No. | SHR9146 | IS | IS-normalized | No. | SHR9146 | IS | IS-normalized |
| --- | --- | --- | --- | --- | --- | --- | --- |
| 1-L-1 | 101.8 | 101.6 | 100.2 | 1-H-1 | 99.5 | 100.8 | 98.8 |
| 1-L-2 | 100.3 | 101.1 | 99.1 | 1-H-2 | 99.2 | 100.1 | 99.1 |
| 1-L-3 | 96.4 | 99.0 | 97.4 | 1-H-3 | 101.3 | 100.8 | 100.5 |
| 2-L-1 | 99.5 | 99.2 | 100.3 | 2-H-1 | 101.9 | 101.8 | 100.1 |
| 2-L-2 | 98.7 | 98.8 | 99.9 | 2-H-2 | 100.7 | 101.4 | 99.3 |
| 2-L-3 | 100.3 | 99.2 | 101.1 | 2-H-3 | 99.8 | 101.6 | 98.3 |
| 3-L-1 | 98.7 | 100.5 | 98.2 | 3-H-1 | 98.0 | 99.7 | 98.3 |
| 3-L-2 | 107.2 | 106.1 | 101.0 | 3-H-2 | 99.8 | 101.4 | 98.5 |
| 3-L-3 | 97.2 | 99.9 | 97.3 | 3-H-3 | 100.1 | 100.8 | 99.3 |
| 4-L-1 | 99.5 | 99.0 | 100.5 | 4-H-1 | 101.3 | 101.8 | 99.5 |
| 4-L-2 | 98.7 | 98.8 | 99.9 | 4-H-2 | 101.6 | 103.0 | 98.6 |
| 4-L-3 | 97.9 | 98.6 | 99.4 | 4-H-3 | 101.6 | 102.2 | 99.4 |
| 5-L-1 | 97.9 | 99.4 | 98.5 | 5-H-1 | 103.3 | 104.9 | 98.5 |
| 5-L-2 | 96.4 | 99.0 | 97.4 | 5-H-2 | 105.1 | 106.1 | 99.0 |
| 5-L-3 | 95.6 | 98.8 | 96.8 | 5-H-3 | 102.7 | 102.8 | 99.9 |
| 6-L-1 | 102.6 | 100.9 | 101.6 | 6-H-1 | 103.6 | 104.2 | 99.4 |
| 6-L-2 | 104.9 | 101.4 | 103.5 | 6-H-2 | 101.6 | 102.0 | 99.6 |
| 6-L-3 | 106.4 | 103.1 | 103.2 | 6-H-3 | 101.3 | 101.8 | 99.5 |
| mean | 100.0 | 100.2 | 99.7 | mean | 101.2 | 102.1 | 99.2 |
| SD | 3.4 | 1.9 | 1.9 | SD | 1.7 | 1.6 | 0.6 |
| RSD | 3.4 | 1.9 | 1.9 | RSD | 1.7 | 1.6 | 0.6 |

^*^: Low concentration was 0.15 μg/g; high concentration was 40 μg/g.

Table S5. Extraction recoveries of SHR9146 and IS in mouse plasma at different concentration levels.

| Component | Levels  (μg/mL) | Mean areas (A) | SD (A) | Sample sizes | Mean areas (B) | SD  (B) | Sample sizes | Extraction recoveries |
| --- | --- | --- | --- | --- | --- | --- | --- | --- |
| SHR9146 | 0.15 | 5.80E+03 | 8.87E+01 | 3 | 5.50E+03 | 3.38E+02 | 6 | 94.8 |
|  | 5 | 1.91E+05 | 1.77E+03 | 3 | 1.85E+05 | 1.20E+04 | 6 | 96.8 |
|  | 40 | 1.51E+06 | 4.54E+04 | 3 | 1.53E+06 | 9.37E+04 | 6 | 101.2 |
| IS | 1 | 1.76E+05 | 5.69E+03 | 9 | 1.72E+05 | 1.06E+04 | 18 | 97.6 |

A. spiked after pretreatment; B. spiked before pretreatment.

Table S6. SHR9146 plasma drug concentration (μg/mL)time (h) data in mice following different doses or routs of administration (*n*=6).

| Dosage (mg/kg) | Sampling time(h) | #1 | #2 | #3 | #4 | #5 | #6 | mean  (μg/mL) | SD |
| --- | --- | --- | --- | --- | --- | --- | --- | --- | --- |
| 5 (i.v.) | 0.083 | 7.880 | 6.501 | 6.135 | 7.070 | 7.712 | 5.854 | 6.859 | 0.834 |
|  | 0.25 | 5.214 | 4.634 | 5.530 | 5.068 | 5.867 | 5.061 | 5.229 | 0.426 |
|  | 0.5 | 4.526 | 3.814 | 3.044 | 3.947 | 3.268 | 3.049 | 3.608 | 0.591 |
|  | 1 | 2.446 | 2.136 | 3.488 | 2.715 | 3.587 | 2.184 | 2.759 | 0.638 |
|  | 2 | 0.498 | 0.653 | 0.503 | 0.385 | 0.588 | 1.003 | 0.605 | 0.215 |
|  | 4 | BLQ | 0.061 | 0.354 | 0.073 | 0.198 | 0.073 | 0.126 | 0.129 |
|  | 6 | BLQ | BLQ | BLQ | BLQ | BLQ | BLQ | / | / |
|  | 8 | BLQ | 0.149 | BLQ | BLQ | BLQ | BLQ | / | / |
|  | 10 | BLQ | BLQ | BLQ | BLQ | BLQ | BLQ | / | / |
|  | 24 | BLQ | BLQ | BLQ | BLQ | BLQ | BLQ | / | / |
| 20 (i.g.) | 0.25 | 4.318 | 4.264 | 10.424 | 7.616 | 13.439 | 4.955 | 7.503 | 3.766 |
|  | 0.5 | 12.205 | 7.513 | 6.779 | 4.648 | 12.863 | 4.841 | 8.142 | 3.582 |
|  | 1 | 4.121 | 1.283 | 4.054 | 9.104 | 4.762 | 4.423 | 4.624 | 2.525 |
|  | 2 | 2.104 | 0.333 | 8.420 | 2.159 | 9.708 | 1.135 | 3.976 | 4.019 |
|  | 3 | 6.753 | 2.669 | 0.821 | 0.263 | 4.318 | 2.858 | 2.947 | 2.374 |
|  | 4 | 0.148 | BLQ | 0.385 | 2.256 | 0.255 | 0.112 | 0.526 | 0.857 |
|  | 6 | BLQ | BLQ | 0.242 | BLQ | 0.281 | BLQ | / | / |
|  | 8 | BLQ | BLQ | 0.194 | BLQ | BLQ | BLQ | / | / |
|  | 10 | BLQ | BLQ | BLQ | BLQ | BLQ | BLQ | / | / |
|  | 24 | BLQ | BLQ | BLQ | BLQ | BLQ | BLQ | / | / |
| 40 (i.g.) | 0.25 | 0.213 | 6.513 | 5.997 | 6.990 | 5.088 | 4.173 | 4.829 | 2.477 |
|  | 0.5 | 9.747 | 9.915 | 0.706 | 5.062 | 0.282 | 6.133 | 5.307 | 4.198 |
|  | 1 | 14.150 | 8.056 | 18.587 | 13.042 | 5.372 | 0.689 | 9.983 | 6.513 |
|  | 2 | 0.719 | 12.276 | 9.415 | 3.103 | 2.389 | 1.618 | 4.920 | 4.745 |
|  | 3 | 2.305 | 1.716 | 1.334 | 0.438 | 0.715 | 0.252 | 1.127 | 0.798 |
|  | 4 | 3.921 | 4.455 | 5.669 | 1.014 | 10.453 | 0.582 | 4.349 | 3.592 |
|  | 6 | 0.545 | 1.856 | 0.492 | BLQ | 3.179 | BLQ | 1.012 | 1.261 |
|  | 8 | BLQ | BLQ | 0.582 | BLQ | 0.331 | BLQ | / | / |
|  | 10 | BLQ | BLQ | BLQ | BLQ | BLQ | 0.293 | / | / |
|  | 24 | BLQ | BLQ | BLQ | BLQ | BLQ | 0.136 | / | / |
| 80 (i.g.) | 0.25 | 4.807 | 6.532 | 2.882 | 9.761 | 7.622 | 5.181 | 6.131 | 2.399 |
|  | 0.5 | 9.320 | 11.869 | 11.106 | 13.915 | 11.362 | 10.320 | 11.315 | 1.555 |
|  | 1 | 12.846 | 19.500 | 0.446 | 20.289 | 23.386 | 0.889 | 12.893 | 10.075 |
|  | 2 | 2.807 | 6.223 | 0.769 | 22.499 | 16.613 | 4.650 | 8.927 | 8.636 |
|  | 3 | 0.671 | 9.313 | 2.798 | 5.034 | 8.033 | 2.193 | 4.674 | 3.424 |
|  | 4 | 4.657 | 22.517 | 0.715 | 5.330 | 19.633 | 0.839 | 8.949 | 9.626 |
|  | 6 | BLQ | 0.088 | BLQ | 6.184 | 7.238 | 0.078 | 2.265 | 3.461 |
|  | 8 | BLQ | 0.090 | 4.871 | BLQ | 2.260 | BLQ | 1.203 | 2.008 |
|  | 10 | 0.068 | 7.993 | 0.486 | 0.080 | 1.174 | 0.415 | 1.703 | 3.108 |
|  | 24 | BLQ | 0.096 | 0.151 | BLQ | BLQ | BLQ | / | / |
| 20 (i.g. bid, 7 days) | Fifth day | BLQ | BLQ | BLQ | BLQ | BLQ | BLQ | / | / |
|  | Sixth day | BLQ | BLQ | BLQ | BLQ | BLQ | BLQ | / | / |
|  | 0 | BLQ | BLQ | BLQ | BLQ | BLQ | BLQ | / | / |
|  | 0.25 | 4.301 | 4.676 | 7.420 | 6.349 | 6.700 | 4.834 | 5.713 | 1.275 |
|  | 0.5 | 4.970 | 8.803 | 11.634 | 11.051 | 5.196 | 6.469 | 8.020 | 2.917 |
|  | 1 | 9.902 | 9.924 | 7.461 | 7.708 | 10.071 | 11.514 | 9.430 | 1.553 |
|  | 2 | 3.968 | 4.754 | 8.083 | 3.763 | 5.945 | 10.521 | 6.172 | 2.657 |
|  | 3 | 2.420 | 2.321 | 7.628 | 3.494 | 1.964 | 1.194 | 3.170 | 2.307 |
|  | 4 | 2.144 | 1.661 | 0.809 | 1.078 | 1.098 | 2.751 | 1.590 | 0.745 |
|  | 6 | 0.363 | 0.277 | 0.481 | 0.091 | 0.141 | 1.368 | 0.453 | 0.470 |
|  | 8 | BLQ | 0.077 | 0.148 | 0.055 | BLQ | 0.068 | 0.058 | 0.055 |
|  | 10 | BLQ | BLQ | 0.058 | BLQ | 0.085 | BLQ | / | / |
|  | 24 | BLQ | BLQ | BLQ | BLQ | 0.054 | BLQ | / | / |

Table S7. SHR9146 concentrations (μg/g) - time (h) data in different mouse tissues (male: *n*=3; female: *n*=3)

| Groups | | male | | | | |  | | female | | | |
| --- | --- | --- | --- | --- | --- | --- | --- | --- | --- | --- | --- | --- |
| **tissues** | **time(h)** | **#1** | **#2** | **#3** | **Mean** | **SD** | **#4** | **#5** | | **#6** | **Mean** | **SD** |
| Stomach | 1 | 39.885 | 32.524 | 41.599 | 38.003 | 4.821 | 31.730 | 24.126 | | 27.411 | 27.756 | 3.814 |
|  | 3 | 0.927 | 18.374 | 11.306 | 10.202 | 8.776 | 28.434 | 24.290 | | 28.997 | 27.240 | 2.570 |
|  | 6 | 1.364 | 0.499 | 1.545 | 1.136 | 0.559 | 1.544 | 2.234 | | 7.838 | 3.872 | 3.452 |
|  | 24 | 0.363 | 0.231 | 0.904 | 0.499 | 0.356 | 0.908 | 0.854 | | 0.782 | 0.848 | 0.063 |
| Adrenal gland | 1 | 24.119 | 30.720 | 15.849 | 23.563 | 7.451 | 48.417 | 30.170 | | 31.257 | 36.615 | 10.235 |
|  | 3 | 0.520 | 13.407 | 11.425 | 8.451 | 6.939 | 35.567 | 15.959 | | 31.830 | 27.785 | 10.411 |
|  | 6 | 0.177 | BLQ | 0.313 | 0.163 | 0.157 | 0.427 | 1.219 | | 4.009 | 1.885 | 1.882 |
|  | 24 | BLQ | BLQ | BLQ | / | / | 0.185 | 0.185 | | 0.214 | 0.195 | 0.017 |
| Small intestine | 1 | 19.156 | 24.690 | 24.120 | 22.655 | 3.044 | 22.755 | 23.199 | | 18.987 | 21.647 | 2.314 |
|  | 3 | 0.411 | 11.992 | 8.326 | 6.910 | 5.919 | 22.446 | 12.038 | | 14.616 | 16.367 | 5.421 |
|  | 6 | 0.117 | BLQ | 0.152 | 0.090 | 0.080 | 0.395 | 0.288 | | 1.815 | 0.832 | 0.852 |
|  | 24 | BLQ | BLQ | BLQ | / | / | BLQ | BLQ | | BLQ | / | / |
| Liver | 1 | 21.953 | 23.893 | 14.099 | 19.982 | 5.186 | 26.622 | 23.393 | | 22.753 | 24.256 | 2.074 |
|  | 3 | 1.367 | 13.792 | 13.853 | 9.671 | 7.191 | 27.066 | 11.815 | | 20.374 | 19.752 | 7.644 |
|  | 6 | 0.702 | 0.351 | 0.845 | 0.633 | 0.254 | 0.621 | 1.090 | | 4.132 | 1.948 | 1.906 |
|  | 24 | BLQ | BLQ | 0.330 | 0.110 | 0.191 | BLQ | 0.058 | | 0.054 | 0.037 | 0.032 |
| Pancreas | 1 | 13.757 | 15.265 | 8.681 | 12.567 | 3.450 | 22.508 | 15.532 | | 15.880 | 17.973 | 3.931 |
|  | 3 | 0.231 | 7.160 | 7.228 | 4.873 | 4.021 | 17.374 | 6.520 | | 13.466 | 12.453 | 5.498 |
|  | 6 | 0.074 | BLQ | 0.127 | 0.067 | 0.064 | 0.089 | 0.150 | | 1.674 | 0.638 | 0.898 |
|  | 24 | BLQ | BLQ | BLQ | / | / | BLQ | BLQ | | BLQ | / | / |
| Lung | 1 | 13.096 | 16.090 | 8.210 | 12.465 | 3.978 | 20.029 | 15.217 | | 13.568 | 16.271 | 3.357 |
|  | 3 | 0.798 | 7.286 | 7.382 | 5.155 | 3.773 | 16.499 | 48.938 | | 12.019 | 25.819 | 20.147 |
|  | 6 | 0.725 | 0.510 | 0.701 | 0.645 | 0.117 | 0.717 | 0.984 | | 2.446 | 1.383 | 0.931 |
|  | 24 | 0.064 | 0.105 | 34.993 | 11.721 | 20.155 | 0.151 | 0.191 | | 0.154 | 0.165 | 0.022 |
| Bladder | 1 | 12.480 | 13.766 | 11.095 | 12.447 | 1.336 | 21.036 | 16.223 | | 13.174 | 16.811 | 3.964 |
|  | 3 | 0.390 | 6.162 | 6.598 | 4.383 | 3.465 | 14.604 | 5.984 | | 10.878 | 10.489 | 4.323 |
|  | 6 | 0.291 | BLQ | 0.199 | 0.163 | 0.149 | BLQ | 0.201 | | 1.685 | 0.629 | 0.921 |
|  | 24 | BLQ | BLQ | BLQ | / | / | BLQ | BLQ | | BLQ | / | / |
| Kidney | 1 | 12.293 | 15.501 | 7.908 | 11.901 | 3.812 | 18.490 | 14.219 | | 13.149 | 15.286 | 2.826 |
|  | 3 | 0.314 | 6.873 | 7.165 | 4.784 | 3.874 | 17.400 | 8.011 | | 12.786 | 12.732 | 4.695 |
|  | 6 | 0.159 | 0.129 | 0.344 | 0.210 | 0.116 | 0.546 | 0.744 | | 3.007 | 1.432 | 1.367 |
|  | 24 | BLQ | BLQ | 0.055 | 0.018 | 0.031 | BLQ | BLQ | | BLQ | / | / |
| Plasma^*^ | 1 | 10.115 | 11.481 | 5.761 | 9.119 | 2.987 | 15.949 | 11.706 | | 11.752 | 13.136 | 2.437 |
|  | 3 | 0.163 | 5.182 | 5.790 | 3.712 | 3.088 | 12.892 | 4.974 | | 9.347 | 9.071 | 3.966 |
|  | 6 | 0.053 | BLQ | 0.094 | 0.049 | 0.047 | 0.070 | 0.124 | | 1.220 | 0.471 | 0.649 |
|  | 24 | BLQ | BLQ | BLQ | / | / | BLQ | BLQ | | BLQ | / | / |
| Heart | 1 | 8.421 | 8.960 | 4.578 | 7.320 | 2.390 | 12.549 | 8.655 | | 7.412 | 9.539 | 2.680 |
|  | 3 | 0.140 | 3.802 | 4.054 | 2.665 | 2.191 | 9.931 | 3.633 | | 7.542 | 7.036 | 3.180 |
|  | 6 | 0.061 | BLQ | 0.085 | 0.048 | 0.044 | 0.052 | 0.098 | | 0.851 | 0.334 | 0.449 |
|  | 24 | BLQ | BLQ | BLQ | / | / | BLQ | BLQ | | BLQ | / | / |
| Skin | 1 | 7.972 | 9.354 | 4.304 | 7.210 | 2.610 | 14.126 | 9.548 | | 9.598 | 11.091 | 2.629 |
|  | 3 | 0.202 | 4.582 | 4.535 | 3.106 | 2.515 | 10.770 | 3.900 | | 8.432 | 7.701 | 3.493 |
|  | 6 | 0.148 | 0.058 | 0.134 | 0.113 | 0.048 | 0.119 | 0.212 | | 1.162 | 0.498 | 0.577 |
|  | 24 | BLQ | BLQ | 0.064 | 0.021 | 0.037 | BLQ | BLQ | | BLQ | / | / |
| Abdominal fat | 1 | 11.412 | 4.018 | 6.182 | 7.204 | 3.802 | 15.163 | 13.658 | | 11.584 | 13.468 | 1.797 |
|  | 3 | 0.268 | 5.628 | 6.299 | 4.065 | 3.306 | 12.161 | 4.954 | | 9.686 | 8.934 | 3.662 |
|  | 6 | 0.067 | BLQ | 0.157 | 0.075 | 0.079 | 0.057 | 0.117 | | 1.072 | 0.416 | 0.569 |
|  | 24 | BLQ | BLQ | BLQ | / | / | BLQ | BLQ | | BLQ | / | / |
| thyroid gland | 1 | 7.253 | 9.009 | 4.759 | 7.007 | 2.135 | 11.884 | 8.433 | | 8.343 | 9.553 | 2.019 |
|  | 3 | 0.163 | 3.897 | 4.136 | 2.732 | 2.228 | 9.783 | 3.848 | | 7.190 | 6.940 | 2.975 |
|  | 6 | 0.071 | BLQ | 0.117 | 0.063 | 0.059 | 0.053 | 0.098 | | 0.983 | 0.378 | 0.524 |
|  | 24 | BLQ | BLQ | BLQ | / | / | BLQ | BLQ | | BLQ | / | / |
| Whole blood^*^ | 1 | 6.818 | 7.419 | 3.946 | 6.061 | 1.856 | 11.230 | 7.393 | | 7.358 | 8.660 | 2.226 |
|  | 3 | 0.119 | 3.297 | 3.895 | 2.437 | 2.030 | 8.869 | 3.336 | | 6.378 | 6.194 | 2.771 |
|  | 6 | BLQ | BLQ | 0.067 | 0.022 | 0.039 | BLQ | 0.079 | | 0.815 | 0.298 | 0.450 |
|  | 24 | BLQ | BLQ | BLQ | / | / | BLQ | BLQ | | BLQ | / | / |
| Skeletal muscle | 1 | 6.096 | 6.868 | 3.823 | 5.595 | 1.583 | 10.584 | 6.380 | | 6.439 | 7.801 | 2.410 |
|  | 3 | 0.111 | 3.247 | 3.299 | 2.219 | 1.826 | 7.825 | 3.125 | | 6.781 | 5.910 | 2.468 |
|  | 6 | BLQ | BLQ | 0.070 | 0.023 | 0.040 | BLQ | 0.071 | | 0.812 | 0.294 | 0.450 |
|  | 24 | BLQ | BLQ | BLQ | / | / | BLQ | BLQ | | BLQ | / | / |
| Spleen | 1 | 5.541 | 5.584 | 3.409 | 4.845 | 1.244 | 8.069 | 5.842 | | 6.193 | 6.701 | 1.197 |
|  | 3 | 0.097 | 2.465 | 2.538 | 1.700 | 1.388 | 6.401 | 2.751 | | 5.069 | 4.740 | 1.847 |
|  | 6 | BLQ | BLQ | 0.051 | 0.017 | 0.029 | BLQ | 0.065 | | 0.659 | 0.241 | 0.363 |
|  | 24 | BLQ | BLQ | BLQ | / | / | BLQ | BLQ | | BLQ | / | / |
| Testis | 1 | 4.163 | 4.834 | 3.007 | 4.001 | 0.924 |  |  | |  |  |  |
|  | 3 | 0.166 | 2.308 | 2.504 | 1.660 | 1.297 |  |  | |  |  |  |
|  | 6 | 0.101 | 0.050 | 0.148 | 0.100 | 0.049 |  |  | |  |  |  |
|  | 24 | BLQ | BLQ | BLQ | / | / |  |  | |  |  |  |
| Brain | 1 | 2.284 | 2.025 | 1.358 | 1.889 | 0.478 | 2.851 | 2.073 | | 2.193 | 2.373 | 0.419 |
|  | 3 | BLQ | 1.018 | 0.981 | 0.666 | 0.577 | 2.082 | 0.887 | | 1.713 | 1.561 | 0.612 |
|  | 6 | BLQ | BLQ | BLQ | / | / | BLQ | BLQ | | 0.261 | 0.087 | 0.150 |
|  | 24 | BLQ | BLQ | BLQ | / | / | BLQ | BLQ | | BLQ | / | / |
| Ovary | 1 |  |  |  |  |  | 21.867 | 16.170 | | 16.988 | 18.342 | 3.080 |
|  | 3 |  |  |  |  |  | 17.161 | 8.006 | | 13.010 | 12.725 | 4.585 |
|  | 6 |  |  |  |  |  | BLQ | 0.270 | | 1.842 | 0.704 | 0.995 |
|  | 24 |  |  |  |  |  | BLQ | BLQ | | BLQ | / | / |
| Uterus | 1 |  |  |  |  |  | 13.423 | 9.041 | | 9.735 | 10.733 | 2.355 |
|  | 3 |  |  |  |  |  | 8.819 | 2.989 | | 6.328 | 6.045 | 2.925 |
|  | 6 |  |  |  |  |  | BLQ | 0.110 | | 0.978 | 0.363 | 0.535 |
|  | 24 |  |  |  |  |  | BLQ | BLQ | | BLQ | / | / |

^*^: Plasma and whole blood concentration (μg/mL)
